# Obesity medication lorcaserin activates brainstem GLP-1 neurons to reduce food intake and augments GLP-1 receptor agonist induced appetite suppression

**DOI:** 10.1016/j.molmet.2022.101665

**Published:** 2022-12-30

**Authors:** Stefan Wagner, Daniel I. Brierley, Alasdair Leeson-Payne, Wanqing Jiang, Raffaella Chianese, Brian Y.H. Lam, Georgina K.C. Dowsett, Claudia Cristiano, David Lyons, Frank Reimann, Fiona M. Gribble, Pablo B. Martinez de Morentin, Giles S.H. Yeo, Stefan Trapp, Lora K. Heisler

**Affiliations:** 1 The Rowett Institute, University of Aberdeen, Aberdeen, UK; 2 Centre for Cardiovascular and Metabolic Neuroscience, Department of Neuroscience, Physiology & Pharmacology, University College London, London, UK; 3 Wellcome-MRC Institute of Metabolic Science-Metabolic Research Laboratories, Medical Research Council Metabolic Diseases Unit, University of Cambridge, Cambridge, UK

**Keywords:** Lorcaserin, Liraglutide, Preproglucagon, Nucleus tractus solitarii, Brainstem, Serotonin 2C receptor

## Abstract

**Objective:**

Overweight and obesity are endemic in developed countries, with a substantial negative impact on human health. Medications developed to treat obesity include agonists for the G-protein coupled receptors glucagon-like peptide-1 (GLP-1R; e.g. liraglutide), serotonin 2C (5-HT_2C_R; e.g, lorcaserin), and melanocortin4 (MC4R) which reduce body weight primarily by suppressing food intake. However, the mechanisms underlying the therapeutic food intake suppressive effects are still being defined and were investigated here.

**Methods:**

We profiled PPG neurons in the nucleus of the solitary tract (PPG^NTS^) using single nucleus RNA sequencing (Nuc-Seq) and histochemistry. We next examined the requirement of PPG^NTS^ neurons for obesity medication effects on food intake by virally ablating PPG^NTS^ neurons. Finally, we assessed the effects on food intake of the combination of liraglutide and lorcaserin.

**Results:**

We found that 5-HT_2C_Rs, but not GLP-1Rs or MC4Rs, were widespread in PPG^NTS^ clusters and that lorcaserin significantly activated PPG^NTS^ neurons. Accordingly, ablation of PPG^NTS^ neurons prevented the reduction of food intake by lorcaserin but not MC4R agonist melanotan-II, demonstrating the functional significance of PPG^NTS^ 5-HT_2C_R expression. Finally, the combination of lorcaserin with GLP-1R agonists liraglutide or exendin-4 produced greater food intake reduction as compared to either monotherapy.

**Conclusions:**

These findings identify a necessary mechanism through which obesity medication lorcaserin produces its therapeutic benefit, namely brainstem PPG^NTS^ neurons. Moreover, these data reveal a strategy to augment the therapeutic profile of the current frontline treatment for obesity, GLP-1R agonists, via coadministration with 5-HT_2C_R agonists.

## 1 Introduction

The prevalence of obesity has reached epidemic levels, with approximately 40% of the adult global population now estimated to be overweight or living with obesity [1]. This has profound health implications because obesity is a major risk factor for multiple chronic diseases. Medications developed to treat obesity include agonists for the G-protein coupled receptors glucagon-like peptide-1 (GLP-1R), serotonin 2C (5-HT_2C_R), and melanocortin4 (MC4R), which all reduce body weight primarily by suppressing food intake. However, the mechanisms underlying the therapeutic food intake suppressive effects of these medications are still being defined. In particular, it is unclear whether the brain circuits targeted by these peripherally administered agonists are distinct entities, or whether their recruited pathways converge to drive weight loss by the potentiation of satiation and/or satiety. To foster precision medicine, a better understanding of the neurophysiological control of appetite is essential to more effectively treat individuals with obesity. Clarifying these circuits may also drive drug discovery efforts for more effective new medications or new combinations of medications.

Consistent with the brain being the master coordinator of energy homeostasis, recent research indicates that the sites of action for the food intake suppressive effects of the GLP-1R agonists, 5-HT_2C_R agonists, and MC4R agonists reside within the CNS [2–9]. However, GLP-1R agonists show limited brain penetrance, at least acutely [2,10,11], and liraglutide-induced cFOS in neurons beyond the circumventricular organs is found primarily in cells not expressing GLP-1Rs [12]. Consequently, GLP-1Rs shielded by the blood—brain barrier may not be the primary direct targets for these drugs [13]. On the other hand, activation of brain GLP-1Rs by centrally-injected GLP-1 or GLP-1R agonists also effectively suppresses feeding, and this is mirrored by the more recent finding that activation of preproglucagon (PPG; product of the *Gcg* gene) expressing neurons in the nucleus of the solitary tract (PPG^NTS^) decreases food intake [14–17]. PPG^NTS^ neurons constitute the primary brain source of GLP-1 [16], however the conditions under which these cells are recruited and their afferent neurocircuitry are poorly understood. In mice, PPG^NTS^ neurons express leptin receptors, and also receive substantial serotonergic input [18–20]. In rats, GLP-1 immunoreactive neurons express 5-HT_2C_Rs, though this has not yet been demonstrated in mice [8]. However, 5-HT receptor agonist treatment in mice illustrates that 5-HT’s effects on PPG^NTS^ neuron activity is both excitatory via the G_q_-coupled 5-HT_2_R family and inhibitory via the G_i_-coupled 5-HT_1A_R subtype [20].

Previous reports indicate that food intake reduction by the 5-HT_2C_R agonist lorcaserin requires a functional brain melanocortin system. Specifically, it was shown that lorcaserin acts via arcuate nucleus of the hypothalamus (ARC) and nucleus of the solitary tract (NTS) pro-opiomelanocortin (POMC) signalling to MC4Rs to decrease food intake [6,7]. POMC’s peptide products α-, β- and γ-melanocyte-stimulating hormone (MSH) are the endogenous agonists for MC4Rs, and activation of MC4Rs effectively decreases food intake [21,22]. Although MC4Rs are expressed in a variety of brain regions, MC4Rs localized within the paraventricular nucleus of the hypothalamus (PVH) have been extensively studied and found to modulate appetite and body weight [3–5]. We previously reported that the expression of 5-HT_2C_Rs exclusively within the NTS and dorsal motor nucleus of the vagus (DMX) is sufficient for lorcaserin’s rapid effect on food intake [6]. Although POMC^NTS^ is required for lorcaserin to produce this acute effect on feeding, the majority of 5-HT_2C_R^NTS^ are not expressed by POMC neurons, thereby providing the possibility that other neurochemically defined neurons may also play a role [6]. Given that PPG neurons are located within the NTS and significantly influence feeding behavior, we hypothesized that 5-HT_2C_R agonists may activate PPG^NTS^ neurons to promote satiation or satiety.

In the present study, we investigated whether there is convergence between GLP-1R-, 5-HT_2C_R-, and MC4R-mediated acute food intake suppression pathways. Building on our previous observation that central and peripheral GLP-1 systems independently suppress eating [17], we assessed the potential for combination therapy utilising systemic GLP-1R agonists and pharmacological activation of PPG^NTS^ neurons for therapeutic suppression of food intake in mice.

## 2 Methods

### 2.1 Mice

Adult male and female *Ppg*^*YFP*^ [23], *Ppg*^*CRE*^ [24], *5-ht*_*2C*_*r*^*CRE*^, *5-ht*_*2C*_*r*^*CRE:YFP*^ [6,25], *Pomc*^*DsRED*^ [6,26], *Mc4r*^*GFP*^ (Jackson Labs, B6.Cg-Tg (*Mc4r*-MAPT/Sapphire)21Rck/J), *loxtb Mc4r null* [4] and wildtype (C57BL/6NJ background from transgenic strains) mice (10–32 weeks old) were used (n = 171). Individual experimental groups included male and female mice unless stated otherwise. Mice were group-housed whenever possible in individually ventilated cages in a temperature-controlled environment (20–22 °C) and maintained on a 12 h light/12 h dark cycle with ad libitum access to chow (Teklad 2018/7912, Envigo or CRM(P) 801722, Special Diet Services) and water unless otherwise stated. All experiments were performed in accordance with the U.K. Animals (Scientific Procedures) Act, 1986 and received local and Home Office ethical approval.

### 2.2 Single nucleus RNA sequencing (Nuc-Seq)

The clustered mouse brainstem Nuc-Seq data (generation described in [27]) is available from NCBI Gene Expression Omnibus Series (GSE168737). Analysis was performed using the Seurat package version 4.0.3 [28,29]. To remove the nuclei with low quality reads, nuclei with less than 1000 reads or 200 features were removed. Additionally, nuclei with more than 6000 features, which may be doublets, were also removed. Nuclei expressing at least one 1 raw UMI count of Gcg gene were extracted for analysis. This subset was then log-normalised and scaled, followed by principal component analysis (PCA). It then underwent non-linear dimensional reduction using T-distributed Stochastic Neighbour Embedding (tSNE) and unsupervised clustering using Louvain algorithm. The violin plots, feature plots and heatmaps were generated using the Seurat package.

### 2.3 Tissue processing

Tissue was processed as previously described [6,30,31]. Briefly, under deep terminal anaesthesia (200 mg/kg pentobarbital i.p.), mice were transcardially perfused with phosphate buffered saline (PBS) followed by 10% neutral buffered formalin (Sigma—Aldrich). Brains were extracted, post-fixed in 10% neutral-buffered formalin at 4 °C, cryoprotected in 30% sucrose in PBS at 4 °C and then sectioned coronally on a freezing sliding microtome at 25 μm. A total of five equal series was collected for each brain. For subsequent immunohistochemistry and in situ hybridization/immunohistochemistry (below), a single series was processed resulting in 125 μm separating sections.

### 2.4 Immunohistochemistry (IHC)

*Ppg*^*YFP*^ NTS tissue was processed free-floating and incubated with chicken anti-green fluorescent protein (GFP; AbCam, ab13970, 1:1000) and/or rabbit anti-cFOS (Calbiochem, PC38, 1:5000) primary antibodies. This anti-GFP antibody binds selectively to yellow fluorescent protein (YFP) in this mouse [32]. YFP was visualized fluorometrically with an anti-chicken Alexa Fluor 488 (1:500, Jackson ImmunoResearch Laboratories) secondary antibody (1:500, Life Technologies) and cFOS chromogenically with a biotinylated donkey anti-rabbit (1:500, Jackson ImmunoResearch Laboratories) and diaminobenzidine (DAB) as previously described [31,33]. For the cFOS study, 10 mice were injected i.p. with either saline or 10 mg/kg lorcaserin and perfuse-fixed with 4% paraformaldehyde (PFA) 2 hr later. Brains were excised and post-fixed overnight in 4% PFA at 6 °C. Brains were stored in PBS until IHC processing.

### 2.5 In Situ hybridization (ISH) and IHC

Fluorescent ISH (FISH) using digoxigenin (DIG)-UTP labelled RNA probes was performed in conjunction with IHC. Specifically, to label *Htr2c* (5-HT_2C_R) mRNA-expressing neurons, a linearized expression vector (pBluescript SK-) containing 3 kb of rat 5-HT_2C_R cDNA (XM_01761920.1: 28–3006) was used as DNA template for *in vitro* transcription [34]. To generate a DNA template for labelling *Ppg* mRNA-expressing neurons, PPG cDNA (NM_008100.2: 136 to 946) was PCR-amplified from mouse brainstem cDNA. In vitro transcription of antisense and sense probes was performed with a DIG-RNA Labelling Mix (Roche, Mannheim, Germany) using either T7 or T3 polymerase.

FISH and IHC in the same tissue were performed with either IHC (free floating) then FISH (NTS sections mounted on slides) or FISH then IHC. In *Pomc*^*dsRED*^ mice, red fluorescent protein (RFP) IHC was performed followed by *Ppg* FISH. In *Mc4r*^*GFP*^ mice, GFP IHC was conducted and then *Ppg* FISH. In *Ppg*^*YFP*^ mice, *Ppg* FISH was performed followed by GFP IHC and *5-ht*_*2C*_*r* FISH was conducted followed by GFP IHC. In *5-ht*_*2C*_*r*^*CRE:YFP*^ mice ARC-injected with anterograde tracer AAV9.CAG.-Flex.tdTomato.WPRE.bGH, *Ppg* FISH was performed followed by red fluorescent protein (RFP) IHC. NTS sections on slides were incubated with 1% sodium borohydride (15 min), 0.1 M Triethanolamine (TEA) (20 min), 0.25% Acetic Anhydride in 0.1 M TEA (10 min) and washed in saline sodium citrate (SSC) buffer [35]. Sections were then pre-incubated in hybridization buffer (1 hr) followed by overnight incubation with DIG-UTP-labelled probes (1 or 2 ng/μl) in hybridization buffer at 72 °C. The next day, sections were washed with 2xSSC and incubated in 20 μg/ml RNase A (30 min), 1xSSC (15 min), 0.1xSSC (30 min), 1% H_2_O_2_ (15 min), wash buffer (100 mM Tris, pH 7.5, 150 mM NaCl; 3 × 5 min) and immersed in a blocking buffer (100 mM Tris, pH 7.5, 150 mM NaCl, 0.5% blocking reagent FP1020, Perkin–Elmer, Boston, USA; 1 h). Sections were then incubated overnight with a peroxidase conjugated anti-DIG antibody (1:200, Roche, Mannheim, Germany) and primary antibodies for GFP/YFP (chicken anti-GFP, 1:1000, ab13970, AbCam, Cambridge UK) or dsRed/tdTomato (Rabbit anti-RFP, 1:500, 600-401-379, Rockland Immunochemicals, Inc, Limerick PA, USA). The following day, the sections were treated with TSA PLUS Biotin Kit (PerkinElmer Inc., Waltham, MA USA) and the respective mRNAs (*5-ht*_*2C*_*r* or *Ppg*) visualized with streptavidin conjugated Alexa Fluor 488 (1:500, S11223, Life Technologies, Carlsbad, CA USA) or 568 (1:500, 1024067, Life Technologies). IHC was performed concomitantly with the FISH detection with anti-rabbit Alexa Fluor 568 (1:500, A10042, Life Technologies, Carlsbad, CA USA) or anti-chicken Alexa Fluor 488 (1:500, 703-545-155, Jackson ImmunoResearch Laboratories) secondary antibodies. Dual ISH and IHC in *Ppg*^*YFP*^ tissue was performed with a ^35^S UTP radio-labelled PPG probe to detect *Ppg* mRNA and a goat α-GFP antibody (Abcam, 1:1000) to visualize YFP as previously described [19,35].

### 2.6 3D rendering of NTS IHC and FISH signal expression

3D models for the distribution of *Ppg*^*YFP*^-IR + *5-ht*_*2C*_*r* mRNA (FISH), *Pomc*^*dsRED*^-IR + *Ppg* mRNA (FISH) and *Mc4r*^*GFP*^-IR + *Ppg* mRNA (FISH) were generated by assembling 11 to 14 consecutive NTS sections spaced 125 μm apart with *Free D* modelling software [36]. The NTS, the surface of the brain and the central canal were outlined for each section, and the anatomical location of individual FISH or IHC labelled cells was entered manually. Colours and cell sizes were assigned in close resemblance to the structures in the digitized images, with single-labelled cells shown in red and green and dual-labelled cells shown in yellow. 3D models are presented in a caudal to rostral orientation.

### 2.7 Ablation of PPG^NTS^ neurons

*Ppg*^*CRE*^ mice were anaesthetised with ketamine hydrochloride (50 mg/kg, i.m.) and medetomidine (1 mg/kg, i.m.) with peri-operative carprofen analgesia (5 mg/kg s.c.). The skull was fixed in a stereotaxic frame and the head ventroflexed to create a right angle between the nose and the neck. Neck muscles were bisected along the midline from the occipital crest to the first vertebrae, and the atlanto-occipital membrane was horizontally bisected to reveal the dorsal brainstem surface and obex. Using a glass microcapillary pulled to a 40 μm tip diameter, 250 nl of virus (AAV8-mCherry-DIO-DTA or AAV8-DIO-EGFP; UNC, ETHZ; Supplementary Table 1) was injected slowly (50 nl/min) bilaterally at the following coordinates relative to obex: 0.50 mm lateral, 0.10 mm rostral, and 0.35 mm ventral. At the end of experiments expression of viral transgenes and successful ablation was confirmed immunohistologically for all experimental animals included in analyses as described previously [16]. PPG cell bodies were absent from the caudal NTS of all AAV8-mCherry-DIO-DTA injected mice, whilst PPG cells in the IRt remained intact in these animals (Figure 2K; Supplementary Figs. 2d–j).

### 2.8 Neuronal projections studies

For anterograde tracer studies, methods were used as previously described [6,37,38]. Briefly, adult male *5-ht*_*2C*_*r*^*CRE*^ mice were anaesthetised with isoflurane (5% induction, 2% maintenance) and given analgesia (carprofen 5 mg/kg, SC) before, during and 48 h after the surgery. Mice were injected unilaterally using a pneumatic injector (Harvard Apparatus, Cambridge, UK) with AAV9.CAG.-flex.tdTomato.WPRE.bGH (Penn Vector Core; Virus titer 1.55e13 GC/ml) using ARC coordinates: 1.58 mm caudal from bregma, 5.75 mm depth from brain surface and 0.2 mm lateral from midline. Brains were collected 3 weeks later and tissue was processed for dsRed-IR and *Ppg* FISH, as detailed above in the ISH and IHC section 2.5.

### 2.9 Feeding studies

The feeding behavior experiments in PPG^NTS^ ablated mice were conducted using a mixed model design. Mice in control (eGFP-transduced) and ablated (DTA-transduced) cohorts each received both saline and test drugs (i.e. lorcaserin and melanotan II [MT-II], respectively) in a counterbalanced order separated by a minimum 72 hr washout period. To prevent any confound from stress-induced hypophagia, mice were habituated to single-housing, handling, saline dosing and the food measurement protocol, such that baseline intakes were stable for ≥3 saline-dosed habituation sessions prior to commencement of test drug sessions. On habituation and test days, mice were weighed and food removed for 3 hr prior to dark onset to entrain mice to eat consistently at dark onset, but without inducing a negative energy balance that would elicit a large refeed response to physiologically recruit PPG^NTS^ neurons [16]. Mice were administered obesity medications or saline vehicle i.p. at 5 mg/ml dose volume 30 min prior to dark onset, at which point a pre-weighed amount of their standard chow (Teklad 2018, Envigo) was returned, and intake measured 1, 2, 4 and 21 hr later, at which point 24 hr body weight change was also measured. Changes in body weight at this 24 hr time point are not expected to reflect changes in body fat, but rather overall changes in daily food intake.

All other feeding studies were performed using a similar design, with intake assessed up to 6 hr, unless otherwise specified. A subset of *Mc4r* wild type control littermates were fed 60%-fat diet (HFD; Test Diet, 58Y1) from 3 weeks of age to match *Mc4r* knockout obesity. All other genotypes were fed chow throughout.

### 2.10 Drugs

Drugs were prepared in sterile saline. Lorcaserin (LGM Pharmaceuticals) was administered at 2.5–10 mg/kg, i.p.; exendin-4 (Tocris) was administered at 0.004–0.04 mg/kg, i.p.; liraglutide (Tocris and Novo Nordisk) was administered at 0.01–0.2 mg/kg, s.c or i.p. and melanotan II (MT-II, Tocris) was injected at 3 mg/kg, i.p.

### 2.11 Statistical analyses

Raw data were processed in Microsoft Excel, plots were generated in GraphPad Prism 7.0 and statistical analyses conducted in SPSS 26.0 (IBM Corp, NY, USA). Food intake data are presented as individual data points, including within-subjects relationships where appropriate, and summary data are presented as mean ± SEM. Food intake data were initially analysed by two- or three-way mixed model ANOVA, with drug treatment as the within-subjects factor and virus (or genotype) and sex as between-subjects factors. No significant interactions with sex were observed. Data from all mice were analysed with treatment x virus (or genotype) mixed model two-way ANOVA or one-way within-subjects ANOVA for lorcaserin dose–eresponse and lorcaserin + liraglutide combination study. Where sphericity could not be assumed for repeated-measures ANOVA, the Geisser-Greenhouse adjustment was applied, in which case the revised fractional degrees of freedom are reported in figure legends. Where treatment × virus interactions were significant (at p < 0.05), simple main effects with Sidak’s correction for multiple comparisons were performed. Where no significant interaction was observed, main effects of treatment were presented. Cell counts for cFOS-IR were analysed by unpaired Student’s t-test (with Welch’s correction as appropriate) or Mann–Whitney test. Statistics are presented in figure legends and data presented as mean ± SEM.

## 3 Results

### 3.1 Characterisation of PPG^NTS^ neurons via Nuc-Seq and histochemistry

**Figure 1:**
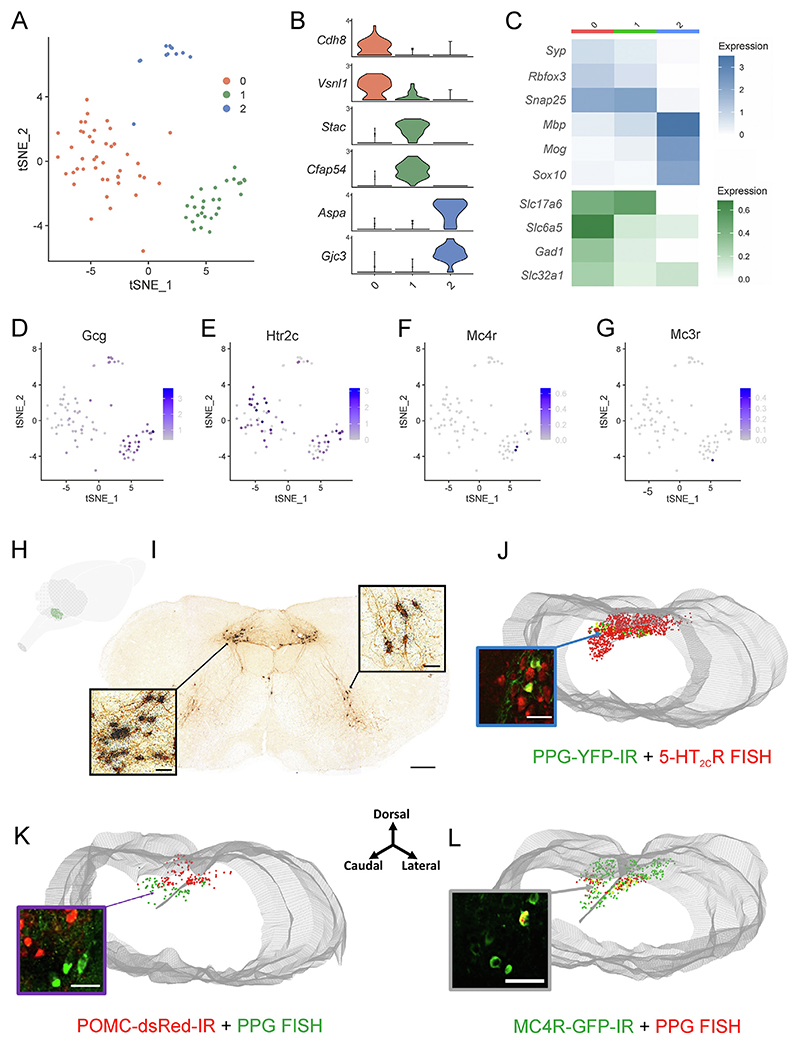
PPG^NTS^ neurons express 5-HT_2C_Rs but not POMC or MC3/4Rs. (A) tSNE plot of 86 preproglucagon (PPG) cells (*Gcg*+) from the brainstem single nucleus RNA sequencing dataset grouped into three clusters. (B) Violin plots showing the relative expression levels of two marker genes for each of the three PPG cell clusters. (C) Heatmaps revealing the average relative expression of canonical neuronal and oligodendrocyte markers within each PPG cell cluster. (D–G) Feature plots showing the relative expression levels of *Gcg* (D) *Htr2c* (E), *Mc4r* (F) and *Mc3r* (G) in the PPG cells. (H) Three-dimensional (3D) schematic of the whole mouse brain illustrating the distribution of PPG neurons (green dots) within the nucleus of the solitary tract (NTS). (I) Representative photomicrograph of brainstem coronal section from a *Ppg*^*YFP*^ mouse processed for immunohistochemistry for GFP-IR (brown stain) and in situ hybridization (ISH) with a ^35^S-labelled *Ppg* riboprobe (black grains) illustrating co-labelling within the NTS and intermediate reticular nucleus (IRt) (n = 3 mice). (J–L) 3D rendering of the caudal to rostral NTS (J) from *Ppg*^*YFP*^ mice processed for IHC for GFP-IR (green cells) and *5-ht*_*2C*_*r* mRNA with fluorescent ISH (FISH; red cells) illustrating single- and dual-labelling (yellow cells); (K) from *Pomc*^*dsRED*^ mice processed for FISH for *Ppg* mRNA (green cells) and dsRed-IR (red cells) illustrating no overlap of POMC and PPG cells; and (L) from *Mc4r*^*GFP*^ mice processed for FISH for *Ppg* mRNA (red cells) and GFP-IR (green cells) illustrating limited overlap (n = 4−5 mice per study). 3V, third ventricle and gray tube is central canal (cc). Scale bars in I–K: 200 μm; insets 20 μm.

To investigate potential overlap between the central GLP-1 system, NTS 5-HT_2C_Rs and MC4Rs, we performed Nuc-Seq from mouse brainstem [27] (Table 1) and histochemical analyses. Nuc-Seq yielded 86 extracted glucagon (*Gcg*) gene expressing cells, of which 74 were neurons. These cells separated into three major clusters, two of which contained neurons with high and low expression of *Gcg*, respectively (Figure 1A-D). The high *Gcg* cluster (n = 28; cluster 1) was excitatory (glutamatergic), which is consistent with previous research indicating overlap between PPG^NTS^ and glutamatergic neurons [39,40]. However, we also identified a cluster of neurons that expressed low levels of *Gcg* transcript (n = 46; cluster 0) and this was of a primarily inhibitory phenotype (GABAergic/glycinergic). To examine whether 5-HT_2C_Rs are positioned to influence the activity of PPG^NTS^ neurons in mice, we evaluated receptor expression patterns in these neurons. *Htr2c* (5-HT_2C_R) expression was widespread in both clusters, with 67% of cluster 0 cells and 79% of cluster 1 cells expressing this receptor (Figure 1E). This high degree of overlap is consistent with a previous report in rats [8]. In contrast, expression of *Mc4r* and *Mc3r* was very low in both clusters with only 3 cells expressing *Mc4r* and only 1 cell expressing *Mc3r* (Figure 1F,G). Furthermore, out of 86 Gcg expressing and 346 *Pomc* expressing cells, only 2 cells co-expressed both genes, demonstrating very limited overlap between these two cell populations. We next examined 5-HT_2C_R^NTS^ cells from Nuc-Seq (Table 1). In accordance with previous studies, we observed widespread expression of *Htr2c*, and a small proportion of these expressed *Pomc* [6]. Analysis of *Glp-1r* neurons revealed that 63% expressed *Htr2c*, but only minor overlap was detected with *Mc4r* (4%) and *Lepr* (12%) (Table 1). These findings suggest that 5-HT_2C_Rs, but not MC4Rs or MC3Rs, are positioned to influence the activity of a large proportion of PPG cells.

We next employed two transgenic mouse lines: *Ppg*^*YFP*^ and *Ppg*^*CRE*^ to further characterise PPG^NTS^ neurons and to confirm the Nuc-Seq data [16,32]. Anatomically, PPG neurons are located within the NTS and adjacent intermediate reticular nucleus (IRt) at the level of the area postrema and extend approximately 400 μm caudally (Figure 1H). Histochemical validation of the *Ppg*^*YFP*^ mouse line was performed using endogenous *Ppg* mRNA. Specifically, radioactive ISH with a ^35^S *Ppg* riboprobe on NTS sections from *Ppg*^*YFP*^ mice revealed that 97.9 ± 1.2% of *Ppg* mRNA positive cells exhibited YFP immunoreactivity (YFP-IR; using an anti-GFP antibody [32]) and 95.8 ± 1.7% of YFP-IR positive cells co-labelled with ^35^S *Pp*g (Figure 1I). In the IRt, 95.5 ± 1.7% of ^35^S *Ppg* positive cells exhibited YFP-IR and 92.9 ± 1.9% of YFP-IR positive cells co-labelled for ^35^S *Ppg* (Figure 1I). This near total overlap between YFP-IR and endogenous *Ppg* mRNA was replicated using fluorescent ISH (FISH) for *Ppg* (Supplementary Figs. 1a–b). These data confirm that the *Ppg*^*YFP*^ mouse line and in situ methods for labelling *Ppg* mRNA may be used interchangeably to visualize PPG neurons.

We then used the *Ppg*^*YFP*^ line to confirm the Nuc-Seq findings. Using FISH for *Htr2c* co-localized with GFP-IR in *Ppg*^*YFP*^ mice revealed that 38.8 ± 2.8% of PPG cells were co-labelled for *5-ht*_*2C*_*r* mRNA (Figure 1J). Though this rate of co-expression is lower than expected based on the Nuc-Seq method, these data corroborate the finding that 5-HT_2C_Rs are positioned to impact a substantial proportion of PPG^NTS^ neurons in mice. In agreement with the transcriptomics analysis (Table 1), 5-HT_2C_R-PPG neurons comprise a small proportion of the total 5-HT_2C_R^NTS^ population, as shown in a three-dimensional (3D) representation of the expression profile within the NTS (Figure 1J). To establish whether this anatomical localization identifies a previously uncharacterized population of 5-HT_2C_R^NTS^ cells, we examined co-expression of *Ppg* mRNA and POMC using dsRed in a *Pomc*^*dsRED*^ reporter line. We observed that PPG and POMC are separate populations of neurochemically-defined NTS cells (Figure 1K), corroborating a previous report utilizing ^35^S *Ppg* in *Pomc*^*GFP*^ mice [19] as well as the transcriptomics data presented here (Table 1). To further examine whether PPG^NTS^ neurons express MC4Rs, we performed *Ppg* FISH in *Mc4r*^*GFP*^ mice. We observed that a small proportion of PPG cells (13.5 ± 1.3%) co-localize with GFP-IR from this MC4R reporter, consistent with our Nuc-Seq data demonstrating *Mc4r* expression in only three of the 86 identified PPG cells (Figure 1L). Taken together, these findings indicate that 5-HT_2C_Rs are positioned to directly influence the activity of approximately one-third to two-thirds of PPG^NTS^ neurons in mice.

### 3.2 Lorcaserin activates PPG^NTS^ neurons *in vivo*

**Figure 2:**
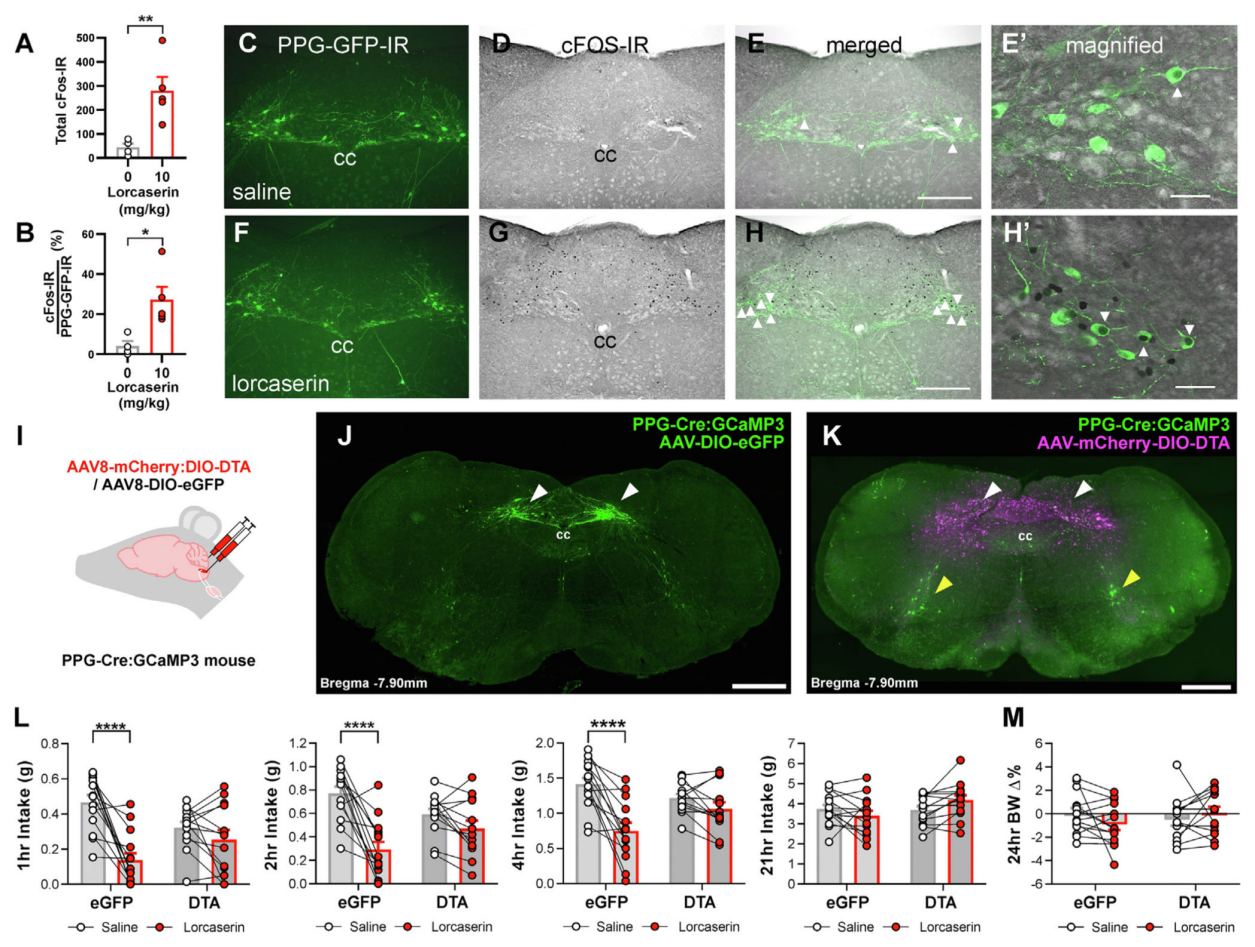
Lorcaserin requires PPG^NTS^ neurons to reduce food intake. (A—H) Lorcaserin-induced (10 mg/kg, i.p.) cFOS immunoreactivity (IR) in PPG^NTS^ neurons in male and female *Ppg*^*YFP*^ mice (n = 10). (A) Lorcaserin increased the number of NTS cFOS-IR cells (*t*_(7)_ = 3.502, p = 0.010) and (B) the percentage of GFP-IR PPG^NTS^ neurons which were cFOS-IR positive (Mann–Whitney *U* = 0, p = 0.0159) compared to saline treated mice. (C—H) Photomicrographs of representative coronal NTS images of GFP-IR (green) and cFOS-IR (black) positive cells following (C—E) saline or (F`—H) lorcaserin treatment. (I) The nucleus of the solitary tract (NTS) of PPG-Cre:GCaMP3 mice was injected bilaterally with either AAV8-mCherry:DIO-DTA to ablate PPG neurons or with AAV8-DIO-eGFP as control. (J,K) Representative photomicrographs showing the presence or absence of PPG^NTS^ neurons (white arrow heads), respectively, after transduction with control or DTA virus. Magenta shows spread of the DTA virus by visualising the Cre-independent transduction of mCherry. Yellow arrowheads indicate PPG^IRT^ neurons that are not reached by the virus. (L) Lorcaserin (7.5 mg/kg i.p.) significantly reduced cumulative dark cycle 1, 2, and 4 h chow intake in male and female eGFP-transduced control (n = 14) but not PPG^NTS^ DTA-ablated (n = 14) *Ppg*^CRE^ mice (1 hr: treatment x virus *F*_(1,25)_ = 10.81, p = 0.0030; 2 hr: treatment x virus *F*_(1,25)_ = 11.93, p = 0.0020; 4hr: treatment x virus *F*_(1,25)_ = 11.35, p = 0.0025). Lorcaserin did not significantly alter (L) 21 hr food intake or (M) 24 hr % change in body weight. cc, central canal. Scale bar in h 200 μm applies to c-h; scale bar in e’ and h’ 50 μm; scale bar in J,K 500 μm. Data are presented as mean ± SEM; *p < 0.05, **p < 0.01, ***p < 0.001.

We next investigated whether systemically administered lorcaserin activates PPG^NTS^ neurons. We first confirmed that 7.5 and 10 mg/kg i.p. lorcaserin significantly reduced chow intake at 1, 3 and 6 hr compared to vehicle treatment in wild type mice (Supplementary Figs. 2a–c). We then determined that lorcaserin (10 mg/kg, i.p.) increased overall NTS neuronal activity compared to saline treatment as measured by cFOS-IR (279 ± 58 cells vs 44 ± 16; Figure 2A and 2C-H’). Furthermore, lorcaserin treatment induced significantly more cFOS-IR specifically in YFP-IR PPG^NTS^ neurons relative to saline treatment (27.3 ± 6.3% vs 4.1 ± 2.5%; Figure 2B). These results demonstrate that a subset of PPG neurons, consistent with the proportion expressing 5-HT_2C_R as determined by FISH, is activated by lorcaserin. This finding extends our previous observation using *ex vivo* brainstem slices which demonstrated that approximately one third of PPG^NTS^ neurons show increased electrical activity upon 5-HT administration, and that this is mediated by 5-HT_2_ receptors [20].

### 3.3 PPG^NTS^ neurons are required for lorcaserin-induced food intake suppression

Having established that a subpopulation of PPG^NTS^ neurons both express 5-HT_2C_Rs and are activated by lorcaserin, we next examined whether these neurons are functionally necessary for the drug’s anorectic effect. A selective loss-of-function approach was employed to ablate PPG^NTS^ neurons in male and female *Ppg*^*CRE*^ mice by injecting either AAV8-mCherry-DIO-Diphtheria toxin subunit A (DTA) or control AAV8-DIO-enhanced green fluorescent protein (EGFP) into the NTS [16,17] (Figure 2I-K). Ablation of PPG^NTS^ neurons was verified in all mice by histological analysis (Supplementary Figs. 2d–j). Chow intake was measured at 1, 2, 4 and 21 hr after dark onset in ad libitum fed mice. Lorcaserin induced a significant reduction in cumulative food intake during hours 1—4 in the control group, however this reduction was prevented by DTA ablation of PPG^NTS^ cells (Figure 2L). Consistent with previous reports, a single dose of lorcaserin was not sufficient to significantly impact 21 hr food intake or 24 hr body weight (Figure 2M; [6]). These findings identify PPG^NTS^ neuron activity as a necessary component of the food intake suppressive effect of lorcaserin in mice and suggest that pharmacological activation of approximately one-third of PPG neurons in the NTS is sufficient to elicit a significant reduction in food intake. Previous observations in rats using intracerebroventricular administration of the GLP-1 receptor antagonist exendin-9 showed a prevention of the anorectic effects of lorcaserin [8], consistent with our conclusion that lorcaserin activates PPG neurons in the brainstem to elicit hypophagia.

### 3.4 PPG^NTS^ neurons are innervated by 5-HT_2C_R^ARC^ neurons and are not required for MC4R agonist food intake suppression

**Figure 3:**
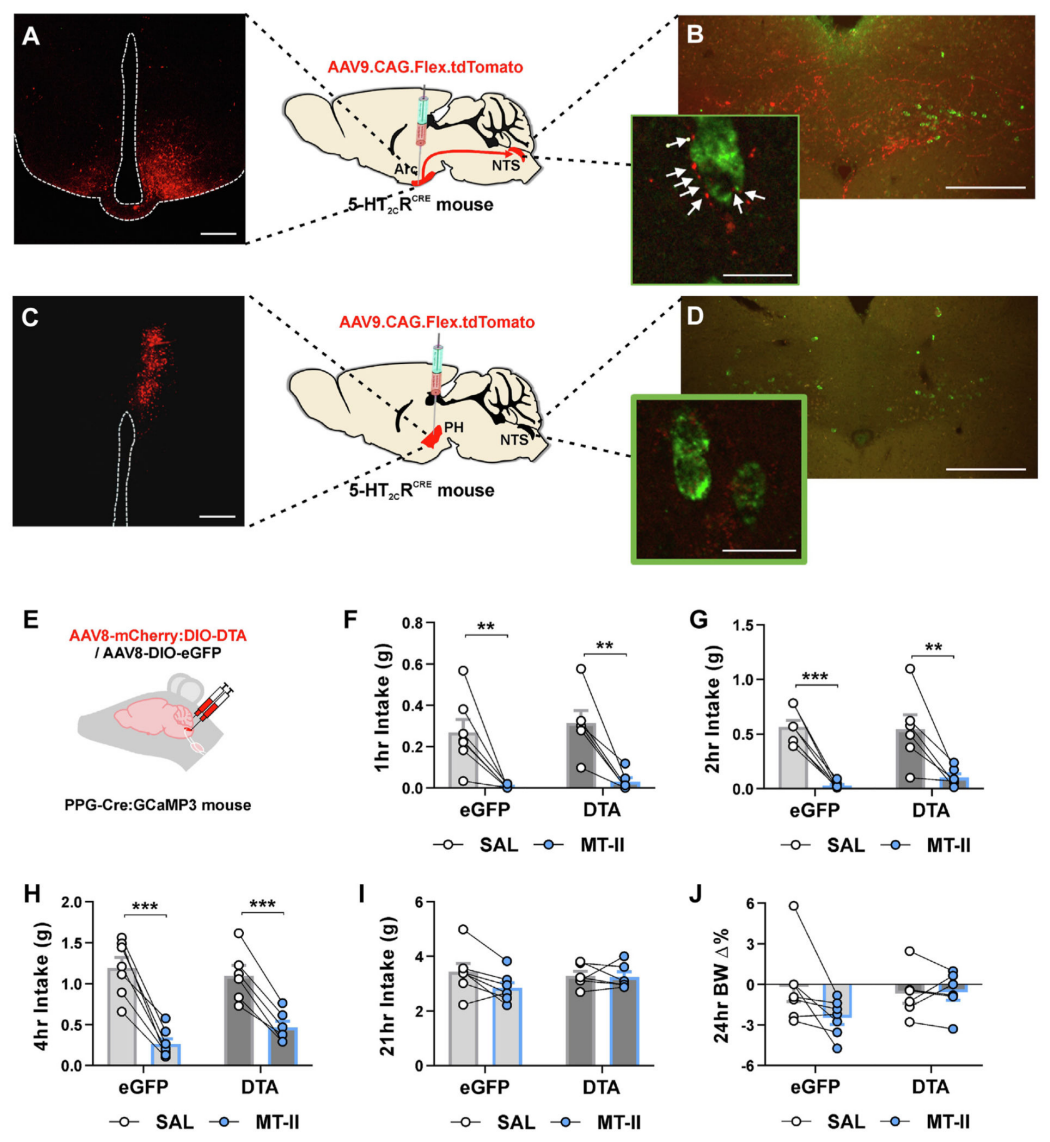
Arcuate nucleus of the hypothalamus 5-HT_2C_R neurons project to PPG^NTS^ neurons and PPG^NTS^ ablation does not affect MC4R agonist hypophagia. (A–D) AAV9.-CAG.Flex.tdTomato.WPRE.bGH (red) injected into hypothalamic subregions of *5-ht*_*2C*_*r*^*CRE:YPF*^ mice (n = 10) to visualize axon terminals within the brain. (A) Arcuate nucleus of the hypothalamus (Arc) injections (n = 4 mice) revealed (B) dense innervation of the nucleus of the solitary tract (NTS) that was not seen following injections outside the Arc (n = 6 mice). Arc derived 5-HT_2C_R terminals (red) were in close apposition to a subset of *Ppg* mRNA neurons (green) vizualized with fluorescent in situ hybridization histochemistry (FISH). (C) Representative photomicrograph of AAV9.CAG.Flex.tdTomato.WPRE.bGH injected into the posterior hypothalamus (PH) and (D) corresponding limited innervation of the NTS (PPG^NTS^ neurons, green). (E–J) Dark onset food intakes and body weight changes in male and female PPG^NTS^ DTA-ablated (n = 6) and eGFP (n = 7) control PPG^Cre^ mice administered saline or preclinical obesity medication MC4R agonist melanotan-II (MT-II; 3 mg/kg i.p.). (E) Schematic showing the injection site of viral delivery into the NTS. MT-II reduced cumulative chow intake in both PPG^NTS^ neuron ablated and control mice at (F) 1 h (treatment F_(1, 11)_ = 38.47, p < 0.0001; treatment x virus *F*_(1,11)_ = 0.04641, p = 0.8334), (g) 2 h (treatment F_(1, 11)_ = 57.07, p < 0.0001; treatment x virus *F*_(1,11)_ = 0.5720, p = 0.4654) and (h) 4 h (treatment F_(1, 11)_ = 108.8, p < 0.0001; treatment x virus *F*_(1,11)_ = 4.105, p = 0.0677). However, MT-II did not significantly reduce intake in either group at (I) 21 h or have an effect on (J) 24 h body weight change. Data are presented as mean ± SEM; *p < 0.05, ***p < 0.001.

The above results, combined with the earlier observation that lorcaserin requires ARC POMC neurons signalling via MC4Rs for its intake suppressive effect [6,7], suggest that lorcaserin exerts feeding effects by recruitment of either parallel or overlapping pathways. Consequently, we explored evidence for crosstalk between the PPG^NTS^ and 5-HT_2C_R/POMC^ARC^⟶MC4R pathway using neuroanatomical tracing and histochemistry to assess whether PPG^NTS^ neurons receive close appositions from 5-HT_2C_R^ARC^ cells. A Cre-inducible AAV9-CAG-DIO-tdTomato tracer virus was injected into the ARC of *5-ht*_*2C*_*r*^*CRE:YFP*^ mice revealed 5-HT_2C_R^ARC^ axons robustly innervate the NTS (Figure 3A-B). Using *Ppg* FISH, we found that 5-HT_2C_R^ARC^ axons were in close apposition to PPG^NTS^ neurons (Figure 3B, inset). This pattern was not observed in *5-ht*_*2C*_*r*^*CRE:YFP*^ mice injected with AAV9-CAG-DIO-tdTomato into another hypothalamic sub-region, the posterior hypothalamus (Figure 3C,D). Within the ARC, the subset of 5-HT_2C_Rs that are both necessary and sufficient to modulate the anorectic effect of 5-HT_2C_R agonists are expressed by POMC neurons [41,42]. POMC peptides signal through the MC4R to impact feeding [43,44], but here we find that PPG neurons show limited *Mc4r* expression (Figure 1). Our anatomical and transcriptomic data therefore suggest that lorcaserin may activate subsets of PPG^NTS^ neurons directly (via PPG^NTS^-5-HT_2C_Rs) and indirectly (via 5-HT_2C_R^ARC^ to PPG^NTS^), but that melanocortin signalling via MC4Rs expressed on PPG neurons may only be a minor contributor to this effect.

We tested this hypothesis by assessing the effect of the α-MSH analogue/MC4R agonist MT-II on food intake and body weight in *Ppg*^*CRE*^ mice with ablated (AAV8-mCherry-DIO-DTA) or functional (AAV8-DIO-EGFP) PPG^NTS^ cells (Figure 3E). Using the same conditions employed in the lorcaserin treatment experiments, we observed that MT-II (3 mg/kg, i.p.) induced a robust anorectic effect over 1–4 h in both control eGFP and PPG^NTS^ ablated mice (Figure 3F-H). The apparently more modest effect of MT-II on 21 h intake and 24 h bodyweight in PPG^NTS^ ablated mice (Figure 3I-J) is most likely a non-specific interaction, driven by a larger post-treatment refeeding response in this group, similar to that previously seen in this ablation model following a prolonged fast [16,17]. Our finding that PPG^NTS^ neurons are not necessary for the acute anorectic effect of MT-II is in line with the small proportion of anatomical overlap and limited expression of MC4R expression on PPG^NTS^ neurons, and with a previous *ex vivo* study employing patch clamp electrophysiology that failed to detect effects of MT-II on the electrical activity of individual PPG neurons [45]. Overall, these findings argue against any substantial direct effect of MC4R activation on PPG^NTS^ neurons, and are consistent with earlier reports that identified the population of MC4Rs that are sufficient to promote MT-II’s effects on 3 h food intake as residing within the PVH [4,5]. Our prior and current findings thus demonstrate that lorcaserin reduces food intake by activating both POMC⟶MC4R neuron driven hypophagia [6,7] and PPG^NTS^ neuron driven hypophagia, and that the loss of either pathway is sufficient to negate the food intake suppressive effects of lorcaserin.

### 3.5 MC4Rs are required for the acute effect of lorcaserin, but not liraglutide or exendin-4

**Figure 4:**
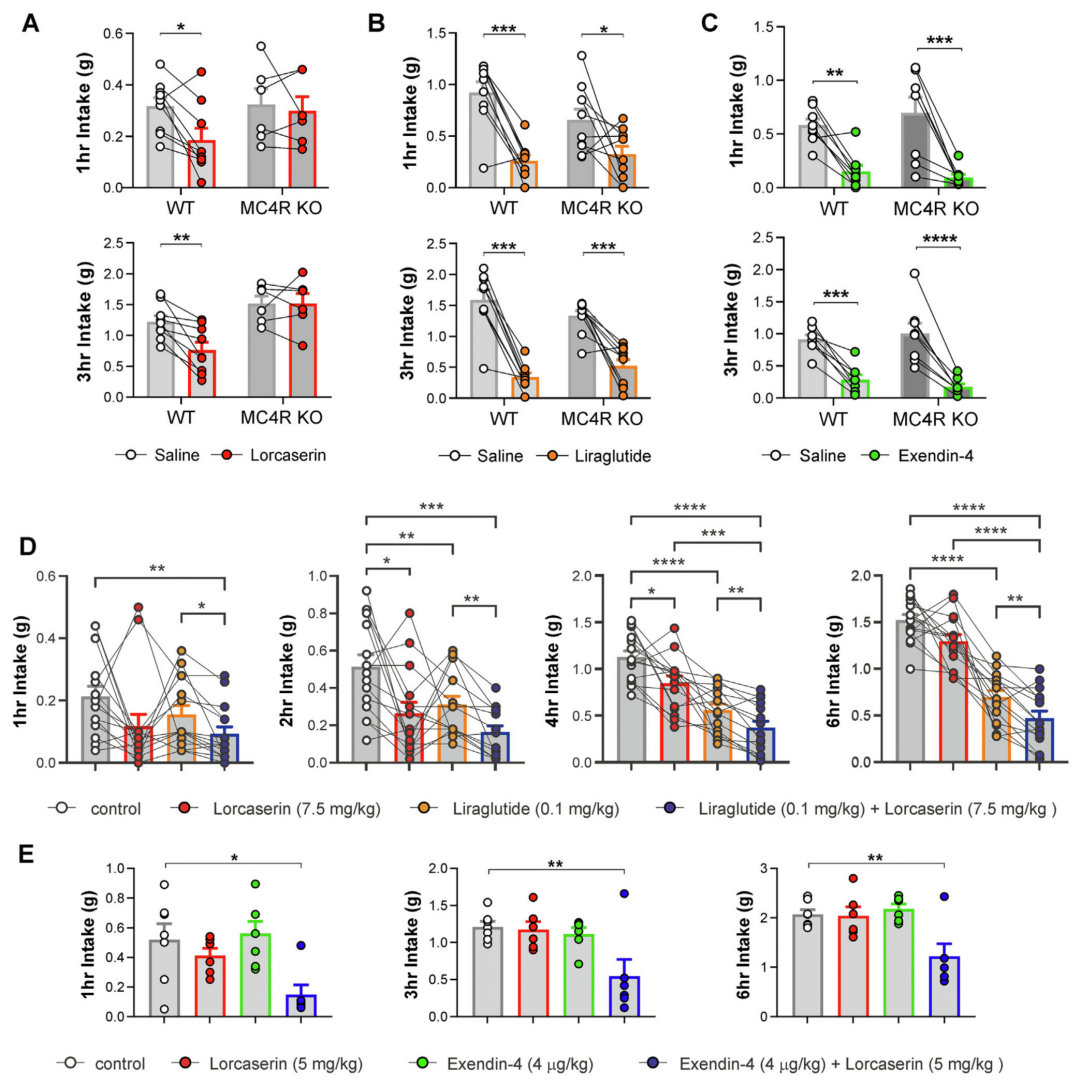
Combination of lorcaserin and GLP-1R agonist produces augmented reduction in food intake. (A) Lorcaserin (7.5 mg/kg, i.p.) signficantly reduced (A) 1 and 3 hr cumulative dark cycle food intake in male and female wild type (n = 9), but not MC4R knockout (n = 7) mice. (1 hr: treatment x genotype F_(1,13)_ = 2.464, p = 0.1405; 3 hr: treatment x genotype F_(1,13)_ = 6.898, p = 0.0209). (B) Liraglutide (0.1 mg/kg, s.c.) significantly reduced 1 and 3 hr cumulative food intake in both adult wild type (n = 8) and *Mc4r* knockout (n = 8) mice (1 hr: treatment x genotype F_(1, 17)_ = 2.932, p = 0.1050; 3 hr: treatment x genotype F_(1, 17)_ = 4.062, p = 0.0600). (C) Exendin-4 (0.04 mg/kg, i.p.) signficantly reduced 1 and 3 hr cumulative dark cycle food intake in both male and female wild type and *Mc4r* knockout mice. (n = 8; 1 hr: treatment F_(1,14)_ = 41.71, p < 0.0001, treatment x genotype F_(1,14)_ = 1.164, p = 0.299; 3 hr: treatment F_(1,14)_ = 84.12, p < 0.0001, treatment x genotype F_(1,14)_ = 1.571, p = 0.231). (D) Individually effective anorectic doses of lorcaserin (7.5 mg/kg, i.p.) and liraglutide (0.1 mg/kg) were tested for their ability to additively suppress food intake over 1–6 hr in male and female wild type (n = 15). Combination treatment produced a greater reduction in food intake compared to lorcaserin and/or liraglutide alone at all timepoints (1 hr: treatment F_(1.7,23.2)_ = 3.943, p = 0.0404; 2 hr: treatment F_(1.8, 25.4)_ = 11.41, p 0.0004; 4 hr: treatment F_(2.2, 30.6)_ = 37.33, p < 0.0001; 6 hr: treatment F_(2.2, 31.4)_ = 73.72, p < 0.0001). Data presented as mean ± SEM; *p < 0.05, **p < 0.01, ***p < 0.001, ****p < 0.0001. (E) Mice were fasted during the dark cycle followed by i.p. injection of saline (n = 7), lorcaserin (5 mg/kg, n = 8), exendin-4 (0.004 mg/kg, n = 6) or a combination of lorcaserin (5 mg/kg) and exendin-4 (0.004 mg/kg) (n = 6). Food intake was measured at 1, 3, and 6 hr. No significant reduction in food intake was measured following treatment with lorcaserin (5 mg/kg) or exendin-4 (0.004 mg/kg) alone at any time point. A significant reduction in food intake following combination treatment was observed at 1 hr (treatment F_(3,22)_ = 4.942, p = 0.009), 3 hr (treatment F_(3,21)_ = 5.288, P = 0.007), and 6 hr (treatment F_(3,21)_ = 6.877, P = 0.002). All data are presented as mean ± SEM; *p < 0.05, **p < 0.01, ***p < 0.001, ****p < 0.0001.

Using a *Mc4r* null line, we confirmed that lorcaserin requires functional MC4Rs to reduce food intake (Figure 4A). We next performed a dose—response study to establish effective doses of the GLP-1R agonists liraglutide and exendin-4 in the reduction of food intake (Supplementary Figs. 3a–f), then assessed the intake suppressing effects of these doses in a *Mc4r* knockout mouse model. In contrast to lorcaserin, GLP-1R agonists liraglutide and exendin-4 did not require functional MC4Rs to impact feeding in mice (Figure 4B-C), which corroborates an earlier report in humans with *MC4R* mutations who were prescribed liraglutide [46].

### 3.6 Co-administration of lorcaserin with liraglutide or exendin-4 produces a greater anorectic effect than either drug individually

Our results indicate that lorcaserin requires PPG^NTS^ neurons and the melanocortin system to influence feeding. In contrast, systemic GLP-1R agonists do not require either PPG^NTS^ neurons or MC4Rs to decrease food intake (17; Figure 4A-C). Based on these findings that suggest the acute hypophagic pathways recruited by lorcaserin and those recruited by systemic GLP-1R agonists are largely separate, we hypothesized that these compounds could be suitable for combination therapy. Therefore, we tested whether combining lorcaserin with liraglutide or exendin-4 could elicit greater reductions in food intake as compared to when these drugs were administered alone. Supporting our hypothesis, combined administration of individually effective doses of lorcaserin and liraglutide resulted in a significantly greater anorectic effect than administration of liraglutide alone at all time points tested (Figure 4D). To investigate whether this effect may be additive or synergistic, we next tested lower doses of lorcaserin and exendin-4 that do not significantly alter feeding when administered as monotherapies. While these doses were ineffective alone, they produced a robust reduction in food intake when administered in combination (Figure 4E). These findings provide compelling support for a dual GLP-1R and 5-HT_2C_R agonist strategy for obesity treatment.

## 4 Discussion

GLP-1R, 5-HT_2C_R and MC4R agonists have been developed for human obesity treatment. However, the mechanisms underlying the therapeutic effects of these medications are still being defined. Here we report that PPG^NTS^ neurons function as a necessary hub through which the 5-HT_2C_R agonist lorcaserin achieves its full therapeutic benefit. Clarifying how lorcaserin produces its therapeutic effect is particularly germane following its withdrawal from the market in the USA due to possible side effects.

These findings are in line with a previous report in rat that brain GLP-1R blockade via exendin-(9–39) prevented brain delivered lorcaserin-induced food intake suppression [8]. Lorcaserin is a 5-HT_2C_R agonist and we identified in an earlier report that 5-HT_2C_Rs exclusively within the NTS and dorsal motor nucleus of the vagus (DMX) are sufficient for lorcaserin’s rapid effect on food intake [6]. Following an examination of neurochemically defined neurons within the NTS, we previously reported that POMC^NTS^ is required for lorcaserin to produce this acute effect on feeding [6,7]. However, the majority of 5-HT_2C_R^NTS^ are not found on POMC neurons, thus other NTS neuron populations could also contribute to lorcaserin anorexia from the NTS. Here we tested this hypothesis. NucSeq analysis revealed that the majority of PPG neurons, the source of brain-derived GLP-1 [16], express 5-HT_2C_Rs. Demonstrating the necessity of PPG neurons in locaserin’s effects, ablation of PPG^NTS^ neurons prevented lorcaserin-induced food intake reduction. Our results taken together with previous research indicate that lorcaserin influences food intake through a complex network of circuitry involving a variety of brain regions and systems. Here we reveal NTS PPG/GLP-1 as a crucial node within this 5-HT_2C_R circuitry.

We also demonstrated limited overlap of circuitry involved in MC4R signalling with that of either peripheral or central GLP-1R signalling. Specifically, we have shown that food intake suppression by the MC4R agonist MT-II does not require intact PPG neurons, and peripherally administered GLP-1R agonists do not require MC4Rs for their anorexic action. Thus, we conclude that both PPG^NTS^ neurons and POMC^NTS/ARC^⟶MC4R neurons are primarily anatomically and functionally independent targets for the anorectic effects of lorcaserin. This lays a clear conceptual groundwork for the combination of GLP-1R agonists and drugs that act via brain PPG neurons and thereby promote brain GLP-1 release to suppress food intake. Our final experiment demonstrated that this rational circuit analysis approach is effective *in vivo* and provides the impetus for future studies to define distinct anorexigenic brain circuits, activation of which could be combined to enhance therapeutic efficacy in obesity treatment.

## 5 Conclusions

The obesity epidemic is a global phenomenon that is having a significant impact on human health. Defining the circuits through which obesity medications promote their therapeutic effects will provide crucial insights into the appropriate choice of medicines for individuals with obesity. For example, patients with total genetic loss of MC4R activity are unlikely to respond well to lorcaserin or the clinical MC4R agonist setmelanotide, but may have a better therapeutic response to liraglutide or semaglutide. All three types of medication would be expected to produce a therapeutic effect in patients with common obesity without genetic alterations in these circuits. Here we identify a necessary mechanism through which obesity medication lorcaserin produces its therapeutic benefit. Importantly, we also provide a strategy to augment the therapeutic profile of the current frontline treatment for human obesity, GLP-1R agonists, via combination with 5-HT_2C_R agonists.

## Figures and Tables

**Table 1:** Quantification of gene expression and co-expression in mouse brainstem cells using single nucleus RNA sequencing (Nuc-Seq).

Cells expressing	Gcg	Pomc	Htr2c	Glp-1r	Mc4r	Lepr
Gcg	86	2	56	1	3	27
Pomc	2	346	215	7	15	45
Htr2c	56	215	6978	113	222	975
Glp-1r	1	7	113	179	7	22
Mc4r	3	15	222	7	289	41
Lepr	27	45	975	22	41	1332

## Data Availability

Data will be made available on request.

## Footnotes

**Appendix A. Supplementary data**
Supplementary data to this article can be found online at https://doi.org/10.1016/j.molmet.2022.101665.
